# Systematic review and network meta-analysis of the accuracy of the orthodontic mini-implants placed in the inter-radicular space by image-guided-based techniques

**DOI:** 10.1186/s12903-023-03079-8

**Published:** 2023-06-12

**Authors:** Fatima Zahrae Mihit Mihit, Álvaro Zubizarreta-Macho, José María Montiel-Company, Alberto Albaladejo Martínez

**Affiliations:** 1 Department of Surgery, Faculty of Medicine and Dentistry, University of Salamanca, 37008 Salamanca, Spain; 2 Department of Implant Surgery, Faculty of Health Sciences, Alfonso X El Sabio University, 28691 Madrid, Spain; 3 Department of Stomatology, Faculty of Medicine and Dentistry, University of Valencia, 46010 Valencia, Spain

**Keywords:** Orthodontics, Micro-screws, Orthodontic anchorage, Mini-implants, Temporary anchorage devices

## Abstract

**Objective:**

The aim of the present systematic review and network meta-analysis (NMA) is to analyze the accuracy of image-guided-based orthodontic mini-implants placement techniques in the inter-radicular space.

**Methods:**

The study was conducted under the PRISMA recommendations. Three databases were searched up to July 2022. In vitro randomized experimental trials (RETs) including static computer-aided implant surgery (s-CAIS), mixed reality (MR), soft tissue static computer-aided implant surgery (ST s-CAIS) and conventional free-hand technique (FHT) for the orthodontic mini-implants placement in the inter-radicular space were selected. The risk of bias was assessed using the Current Research Information System scale. A random effects model was used in the NMA. Direct comparisons were combined with a random effects model in a frequentist NMA to estimate indirect comparisons, and the estimated effect size of the comparisons between techniques were analyzed by difference of means. Inconsistency was assessed with the Q test, with a significance level of *p* < 0.05, and a net heat plot.

**Results:**

A total of 92 articles was identified, and 8 RETs (8 direct comparisons of 4 techniques) were included in the NMA, which examined 4 orthodontic mini-implants placement techniques: s-CAIS, MR, ST s-CAIS, and FHT. Taking FHT as reference, s-CAIS and ST s-CAIS showed statistically significant coronal and apical deviation. In addition, s-CAIS showed statistically significant angular deviation. However, MR did not show statistically significant differences with respect to FHT, which presented the highest p-score. At the coronal deviation, ST s-CAIS presented the highest P-score (0.862), followed by s-CAIS (0.721). At the apical deviation, s-CAIS presented the highest P-score (0.844), followed by ST s-CAIS (0.791). Finally, at the angular deviation s-CAIS presented again the highest P-score (0.851).

**Conclusions:**

Within the limitations of this study, it was found that the image-guided-based orthodontic mini-implants placement techniques showed more accuracy than the free-hand conventional placement technique; specially the computer-aided static navigation techniques for the orthodontic mini-implants placed in the inter-radicular space.

## Background

The use of mini-implants in orthodontic for dental anchorage was firstly introduced by Kanomi, in 1997 [1]; nowadays, they have been widely used in orthodontics due to the clinical versatility and advantages comparing to traditional orthodontic appliances, including a greater comfort for patients, smaller size, immediate loading, less anatomical limitations and low cost [2]. Therefore, orthodontic mini-implants have been widely recommended for many clinical applications, mostly to improve dental anchorage by increasing dental anchorage and decreasing reactive forces [3]. Moreover, its effectiveness has been also observed in expansion procedures that combine palatine mini-implants with indirect anchorage [4]. However, the use of orthodontic mini-implants in the inter-radicular space can lead to clinical complications, being root damage and maxillary sinus perforation the most common risks during mini-implants insertion [5].

Therefore, the choice of the insertion site is essential to minimize the complications and risks of mini-implant insertion. In addition, the orthodontic mini-screw placement site has been analysed by several methods to plan pre-operatively the insertion site of orthodontic micro-screws preventing root contact. The conventional wire-guide, in conjunction with periapical radiographs was a fairly used method [4]. However, radiographic procedures to design surgical guides based on cone-beam computed tomography (CBCT) scan [6], has overcome many of the limitations of 2-dimensional (2D) images. The recently developed CBCT has overcome many of the limitations of conventional dental radiographic by providing better visualization of structures, minimizing blurring, and overlapping of adjacent teeth. They also have reduced cost and significant reduction of radiation [7].

As well as some planning software’s, specifically augmented reality technology and computer-aided static navigation technique, have been compared with conventional freehand technique; having as results that concluding that these new techniques influence the accuracy of orthodontic micro-screws placement in the inter-radicular space and results in fewer intra-operative complications. However, these protocols could be useful, but they are still under study and 100% precision has not been achieved.

The aim of the present study is to conduct a systematic review and network meta-analysis (NMA) to analyze the accuracy of image-guided-based orthodontic mini-implants placement techniques for the orthodontic mini-implants placed in the inter-radicular space.

## Methods

### Study design and registration

This systematic review and network meta-analysis were conducted in accordance with the Preferred Reporting Items for Systemic Reviews and Meta-Analyses (PRISMA, http://www.prisma-statement.org, accessed on 30 July 2020) guidelines. The review also met the PRISMA 2009 Checklist criteria [8]. The registration number is INPLASY202260025 (https://doi.org/10.37766/inplasy2022.6.0025).

### Literature search process

The PICO (population, intervention, comparison, and outcome) question was, ‘What is the efficacy of image-guided-based orthodontic mini-implant placement techniques?’ with the following components: population: orthodontic mini-implants placed in the inter-radicular space by image-guided-based techniques; intervention: orthodontic mini-implants performed through image-guided-based placement techniques; comparison: orthodontic mini-implants performed through static navigation systems, mixed reality techniques and free-hand conventional technique; and outcome: lineal and angular accuracy of orthodontic mini-implants performed through image-guided-based placement techniques. Researchers conducted a search covering all international published literature up to May 2022 using the PubMed, Scopus, and Web of Science databases. The search used the following medical subject heading (MeSH) terms: “orthodontic”, “mini-implant”, “miniscrew”, “surgical guide”, “placement”, “deviation”, “accuracy”. The Boolean operators applied were OR and AND. The terms of the search were structured as follows: ((mini-implant [Title/Abstract]) OR (miniscrew[Title/Abstract])) AND (orthodontic[Title/Abstract]) AND ((surgical guide[Title/Abstract]) OR (placement[Title/Abstract])) AND ((deviation[Title/Abstract]) OR (accuracy[Title/Abstract])). Two researchers (F.M. and A.Z.M.) both carried out the same database search independently of each other. The inclusion and exclusion criteria were applied to titles and abstracts. One researcher (A.A.M.) collected data on the relevant variables. F.M. conducted the systematic review, and two researchers (A.Z.M. and J.M.M.C.) performed the subsequent meta-analysis; these researchers were not involved in the selection process.

### Inclusion and exclusion criteria

The following were the inclusion criteria for the selected studies: in vitro randomized experimental trial (RET). Language and year of publication were not considered as inclusion criteria. Exclusion criteria: randomized clinical trials (RCT), case series (CS), clinical trials (CT), systematic literature reviews, editorials, and clinical cases.

### Data extraction

Independent reviewers (A.Z.M. and A.A.M.) collected the following data from each study: title, author and year of publication, journal in which the article was published, sample size (n), study type, type of image-guided-based placement technique: static computer-aided implant surgery (s-CAIS), mixed reality (MR), soft tissue static computer-aided implant surgery (ST s-CAIS) and conventional free-hand technique and mean and standard deviation for coronal, apical and angular deviation of orthodontic mini-implants. If the independent reviewers disagreed, they consulted a third reviewer (J.M.M.C.).

### Risk of bias

The Current Research Information System (CRIS) scale for methodological quality assessment was used to evaluate the risk of bias of the in vitro studies selected for review. The CRIS scale is made up of four items that assess sample preparation and handling, allocation sequence and randomization process, whether evaluators were blinded, and statistical analysis. Good quality studies were those with information about all variables; fair quality studies had information about two to three variables; and studies were of poor quality if none or only one of the aspects was covered [9].

### Data synthesis and statistical analysis

Meta-analyses were performed using the random effects model to estimate the mean coronal, apical, and angular deviations from the ideal position for the four techniques. Heterogeneity was assessed by the Q test, with a significance level of *p* < 0.1, and with the I^2^.

The net meta-analysis was conducted using a random effects model to estimate the accuracy of image-guided-based placement techniques compared to the free-hand conventional technique. Direct comparisons were combined with a random effects model in a frequentist NMA to estimate indirect comparisons. The NMA was represented using a network graph (four techniques and four designs). Difference of means was used to analyze the estimated effect size of the comparisons between techniques. Forest plots were used to represent comparisons between the techniques (MR, s-CAIS and ST s-CAIS) compared to the free-hand conventional technique (FHT).

Heterogeneity (within designs) and inconsistency (between designs) was assessed by the Q test, with a significance level of *p* < 0.05, and a net heat plot was created to detect possible sources of inconsistency of the direct estimates to the NMA [10].

The four techniques were ranked from a scale of 0 to 1 using a P-score to measure the degree of certainty and determining whether one orthodontic mini-implant placement technique was superior to another [11].

R software and the Metamean and Netmeta statistical package were used to perform the meta-analysis and net-work meta-analysis.

## Results

### Results of the search process

**Fig. 1:**
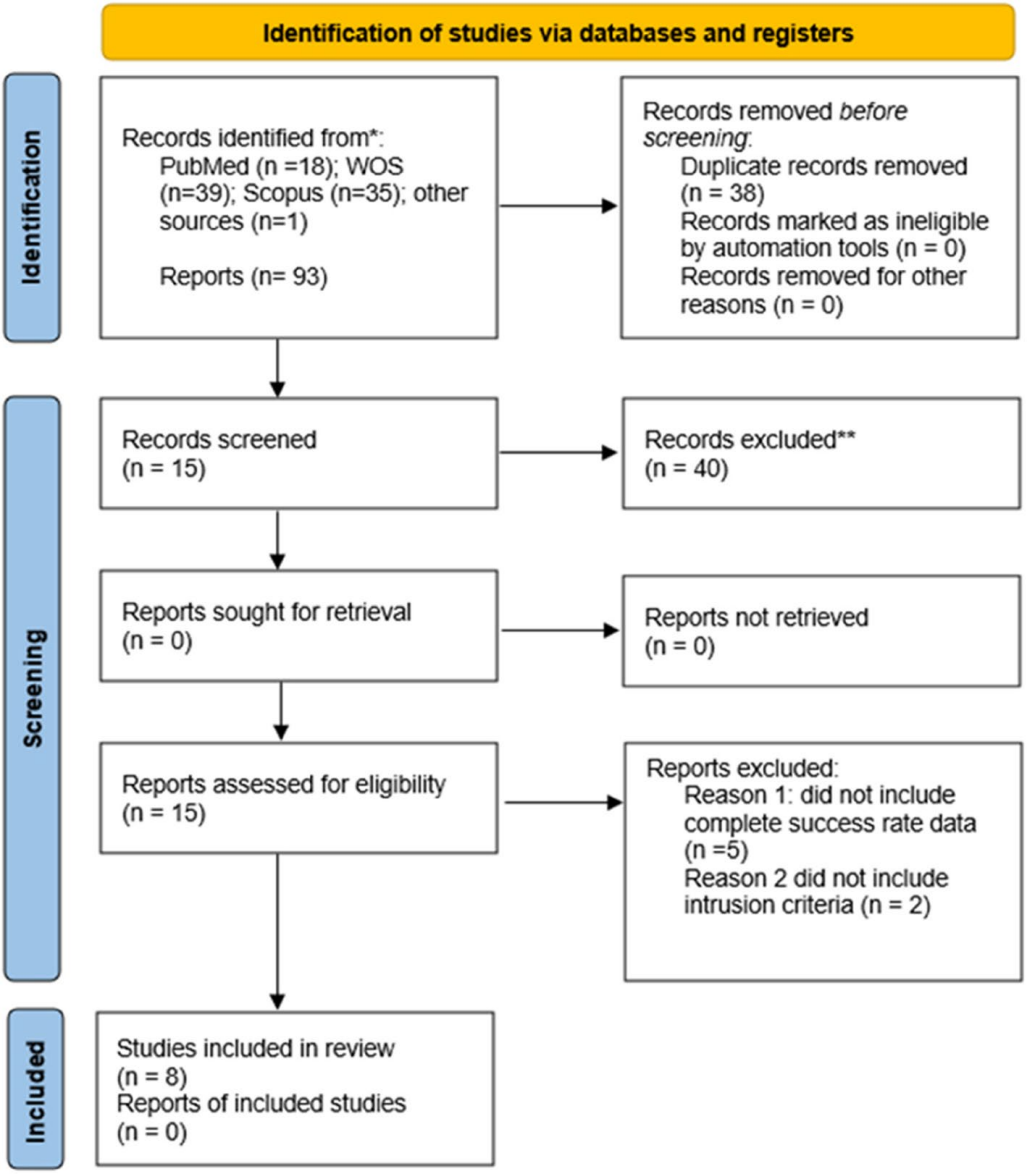
Preferred Reporting Items for Systematic Reviews and Meta-Analyses (PRISMA) flow diagram

The systematic electronic search identified 18 articles in PubMed, 39 in Web of Science (WOS), 35 in Scopus. Of the 92 articles, 56 were discarded as duplicates using RefWorks (https://refworks.proquest.com/reference/upload/recent/, accessed on 10 July 2022). After reading the titles and abstracts, an additional 42 articles were eliminated because they did not fulfil the inclusion criteria. Finally, 8 articles were included in the qualitative and quantitative synthesis because they included all of the required data and variables (Fig. 1).

### Qualitative analysis

4 articles included in the NMA were RETs which analyzed the accuracy tooth-supported static computer-aided implant surgery technique (s-CAIS) [12–15], 3 also assessed the accuracy of soft tissue-supported static computer-aided implant surgery technique (ST s-CAIS) [16–18] and one study also compared the accuracy of a mixed reality appliance technique [19]. Among them, 6 studies analyzed the deviation values using tridimensional cone-beam computed tomography (CBCT) scan [12, 14–16, 18, 19], one study assessed the deviation values using bidimensional periapical radiographies [13] and 1 study analyzed the deviation values using lateral cephalometry [17]. Most of the studies presented a sample size of approximately 8–20 patients, although the sample size ranged from 8 [15] to 180 [13]. The authors of the selected articles compared the mean coronal, apical and angular deviation of orthodontic mini-implants respect to a successful insertion planning. Specifically, Yu et al. planned the successful insertion in the middle of the interradicular space or between the mesial and distal root, and the vertical point was the center of the root length. The vertical angulations to the long axis of adjacent teeth were determined at 30°, 45°, 60°, 75° [16]. Riad Deglow et al. planned the virtual orthodontic micro-screws insertion to a depth of 6 mm, an insertion angle of 90° to the longitudinal axis of the teeth, and a depth of 6.0 mm with respect to the cortical plate. [20] Bae et al. [13] used the successful insertion principles stablished by Lim et al. [20] Suzuki et al. used a software that automatically generates a virtual bisecting line using the cementoenamel junction as a reference, a tangent is drawn to the point of greatest convexity on the proximal root surface of adjacent teeth at the implant site. These lines are extended coronally to form an angle, which is then bisected. The virtual bisecting line thus formed is used as a reference for the optimum planned implant position. The equidistant position between the roots of the adjacent teeth is considered the safest site for mini-screw placement and thus serves as the “gold standard” [14]. Möhlhenrich et al. established a distance between implants of 8 mm, an angle of insertions about 70–80° to the occlusal plane and the height of the mini-implant heads was slightly above the resistance center of the molars [17, 18]. Kniha et al. used the successful insertion position stablished by Möhlhenrich et al. [17, 18] Subsequently, the orthodontic mini-implant placement techniques more near to the peroperative therapeutic planning were considered as more accurate. The results are presented in Table 1.

**Table 1:** Qualitative analysis of articles included in systematic review

Author/Year	Study Type	Sample (n)	Medition Procedure	Study Group	Coronal Deviation		Apical Deviation		Angular Deviation		Complications
					**Mean (mm)**	**DS**	**Mean (mm)**	**SD**	**Mean (°)**	**SD**	
Qiu et al., 2012 [5]	RET	10	CBCT	FHT	0.48	0.46	0.81	0.61	7.49	6.09	10 roots contact
		20		s-CAIS	0.15	0.09	0.28	0.23	1.47	0.92	0 root contact
Yu et al., 2012 [6]	RET	32	CBCT	TOTAL	NAV	NAV	NAV	NAV	1.01	7.25	NAV
		8		s-CAIS at 30º	NAV	NAV	NAV	NAV	1.26	5.19	NAV
		8		s-CAIS at 45º	NAV	NAV	NAV	NAV	1.02	6.89	NAV
		8		s-CAIS at 60º	NAV	NAV	NAV	NAV	-3.1	4.51	NAV
		8		s-CAIS at 75º	NAV	NAV	NAV	NAV	4.8	10.08	NAV
Riad Deglow et al., 2021 [20]	RET	69	CBCT	s-CAIS	1.06	0.59	1.11	0.77	4.66	3.65	0 root perforations
		69		MR	1.74	0.52	1.86	0.65	5.55	2.46	0 root perforations
		69		FHT	2.2	2	1.69	0.82	7.58	3.6	12 root perforations
Bae et al., 2013 [3]	RET	25	CBCT	s-CAIS	0.73	NAV	0.73	NAV	3.14	NAV	16% root contact 0%root damage
		20		FHT	1.56	NAV	1.28	NAV	9.57	NAV	30% root contact 20% root damage
Suzuki et al., 2008 [4]	RET	180	Periapical	conventional s-CAIS	0.6	0.4	2	0.4	1.8	0.9	NAV
		20		3d impression s-CAIS	1	0.4	5.3	1.1	16.9	2.6	NAV
		20		FHT	3.6	1.4	1.5	3.5	21.2	2.9	NAV
Möhlhenrich et al., 2020 [7]	RET	10	CBCT	s-CAIS		3.8	5.5	0.9	51.3	5.8	NAV
		20			4.9	2.9	5.2	1.5	53.2	8	NAV
		10		ST s- CAIS	3.1	3.5	5.4	1.1	58.8	7.1	NAV
		20			2.3	3.2	5.2	1	61.4	10.3	NAV
Möhlhenrich et al., 2019 [8]	RET	20	Lateral CEPH	s-CAIS	0.88	1.65	NAV	NAV	3.6	2.89	NAV
		20		ST s- CAIS	1.65	1.03	NAV	NAV	4.06	3.04	NAV
Kniha et al., 2020 [19]	RET	20	CBCT	s-CAIS	1,47	0,86	1,77	0,85	2,81	2,69	NAV
		20		ST s- CAIS	1,31	0,61	1,91	0,79	6,22	4,26	NAV

*RET* Randomized experimental trial, *CBCT* Cone-beam computed tomography, *s-CAIS* Static computer-aided implant surgery, *ST s-CAIS* Soft tissue static computer- aided implant surgery, *FHT* Free hand technique, *MR* Mixed Reality, *Lat. Ceph* Lateral cephalometry, *NAV* Not available

### Assessment of risk of bias

The methodological quality results were assessed using the CRIS scale are shown in Table 2. Two articles [14, 17] obtained scores of 4, five article obtained the score of 3 [12, 15, 16, 18, 19]; indicating high methodological quality and only one article obtained the score of 2 [13]. Most of selected studies showed a low risk of bias related to the randomization process and blinding procedure.

**Table 2:** Assessment of methodological quality according to the Current Research Information System (CRIS) scale

Author/Year	Sample Preparation and Handling	Allocation Sequence and Randomization Process	Whether the Evaluators Were Blinded	Statistical Analysis	Score
Qiu et al., 2012 [5]	Yes	Yes	Yes	Yes	4
Yu et al., 2012 [16]	Yes	No	Yes	Yes	3
Riad Deglow et al., 2021 [20]	No	Yes	Yes	Yes	3
Bae et al., 2013 [13]	Yes	No	Yes	Yes	3
Suzuki et al., 2008 [14]	Yes	No	No	Yes	2
Möhlhenrich et al., 2020 [17]	Yes	No	Yes	Yes	3
Möhlhenrich et al., 2019 [18]	Yes	Yes	Yes	Yes	4
Kniha et al., 2020 [19]	Yes	Yes	No	Yes	3

### Quantitative analysis results

To determine the mean deviations of each technique from the ideal position at the coronal, apical, and angular axis, 12 meta-analyses were performed using the random effects combination model. All meta-analyses have shown heterogeneity. The techniques that have estimated the least coronal deviation have been s-CAIS with 0.88 mm and ST s-CAIS with 1.42 mm. Regarding the apical deviation, s-CAIS presents the lowest value with 1.21 mm, likewise it presents the lowest angular deviation with a mean estimate of 2.79 degrees (Table 3).

**Table 3:** Metaanalysis of the deviations of each orthodontic mini-implant placement technique from the ideal position

		**Mean**	**95%-CI**	**Z value; p-valor**	**K = studies; ** ***n*** ** = sample**	**Q value; p-valor, I**^**2**^
**Coronal Deviation** **(mm)**	***FHT***	2,08	0,28–3,88	Z = 2,26; *p* = 0,024	K = 3 (5,14,20); *n* = 79	Q = 99,7; *p* < 0,001; 97,9%
	***MR***	1,72	1,59–1,84	Z = 27,4; *p* < 0,001	K = 1 (20); *n* = 20	Q = 0; *p* = 1; 0%
	***s-CAIS***	0,88	0,19–1,56	Z = 2,51; *p* = 0,012	K = 4 (5,18,19,20); *n* = 129	Q = 197; *p* < 0,001; 98,4%
	***ST s-CAIS***	1,42	1,11–1,74	Z = 8,80; *p* < 0,001	K = 2 (18,19)	Q = 1,61; *p* = 0,204; 38,1%
**Apical Deviation** **(mm)**	***FHT***	1,30	0,56–2,05	Z = 3,42; *p* = 0,001	K = 3 (5,14,20); *n* = 79	Q = 16,5; *p* < 0,001; 87,9%
	***MR***	1,86	1,70–2,01	Z = 23,7; *p* < 0,001	K = 1 (20); *n* = 20	Q = 0; *p* = 1; 0%
	***s-CAIS***	1,21	0,18–2,24	Z = 2,31; *p* = 0,021	K = 4 (5,18,19,20); *n* = 129	Q = 879,8; *p* < 0,001; 99,6%
	***ST s-CAIS***	1,31	1,04–1,57	Z = 9,60; *p* < 0,001	K = 1(19); *n* = 20	Q = 0; *p* = 1; 0%
**Angular Deviation** **(º)**	***FHT***	12,1	1,73–22,5	Z = 2,28; *p* = 0,022	K = 3 (5,14,20); *n* = 79	Q = 309; *p* < 0,001; 99,4%
	***MR***	5,55	4,97–6,13	Z = 18,7; *p* < 0,001	K = 1 (20); *n* = 20	Q = 0; *p* = 1; 0%
	***s-CAIS***	2,79	1,76–3,82	Z = 5,32; *p* < 0,001	K = 5 (5,14,18,19,20); *n* = 149	Q = 53,2; *p* < 0,001; 92,5%
	***ST s-CAIS***	5,04	2,93–7,14	Z = 4,69; *p* < 0,001	K = 2 (18, 19); *n* = 40	Q = 3,41; *p* = 0,065; 70,6%

#### Coronal deviation

Eight RETs (eight pairs of comparisons) were included in a frequentist NMA examining four orthodontic mini-implant placement techniques (s-CAIS, ST s-CAIS, MR and FHT) to analyze the accuracy of the orthodontic mini-implants placement techniques at the coronal entry-point. The data were combined with a Random effects model.

**Fig. 2:**
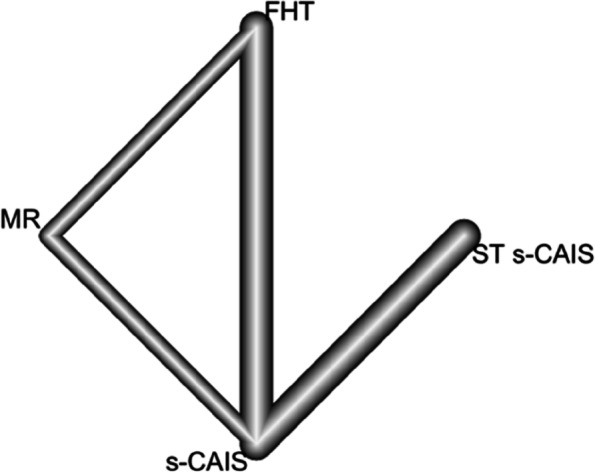
NETWORK plot of orthodontic mini-implant placement techniques at the coronal entry-point. Eight pairs of comparisons between four orthodontic mini-implant techniques and four direct comparisons included

The nodes represent treatments, and the lines connecting the nodes are the four direct comparisons included in the NMA (Fig. 2).

**Fig. 3:**
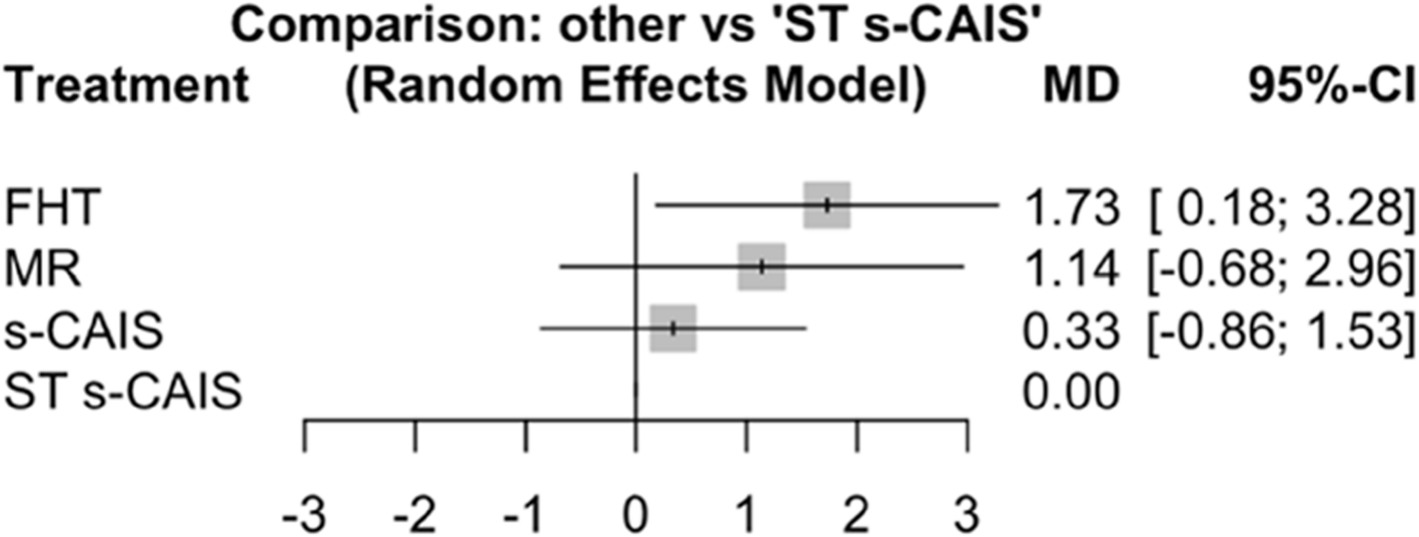
Forest plot of the mean differences (MD) between MR, s-CAIS and ST s-CAIS orthodontic mini-implants placement techniques respect the conventional free-hand technique at coronal entry-point. Column 1 lists the orthodontic mini-implants placement techniques included in the meta-analysis. Column 2 is the forest plot itself, the graphic part of the representation. It plots the coronal deviation between the orthodontic mini-implants placement techniques on both sides of the null effect line, represented by the conventional free-hand technique. Column 3 describes the mean deviation of each orthodontic mini-implants placement techniques respect the conventional free-hand technique, and column 4 presents the confidence interval

Mean differences between MR, s-CAIS and ST s-CAIS orthodontic mini-implants placement techniques and conventional free-hand technique were assessed at coronal entry-point. Taking as reference technique ST s-CAIS, the highest mean difference coronal deviation was FHT (*p*-value = 0.029) followed by MR (*p*-value = 0.220) and s-CAIS (*p*-value = 0.582) (Fig. 3).

**Fig. 4:**
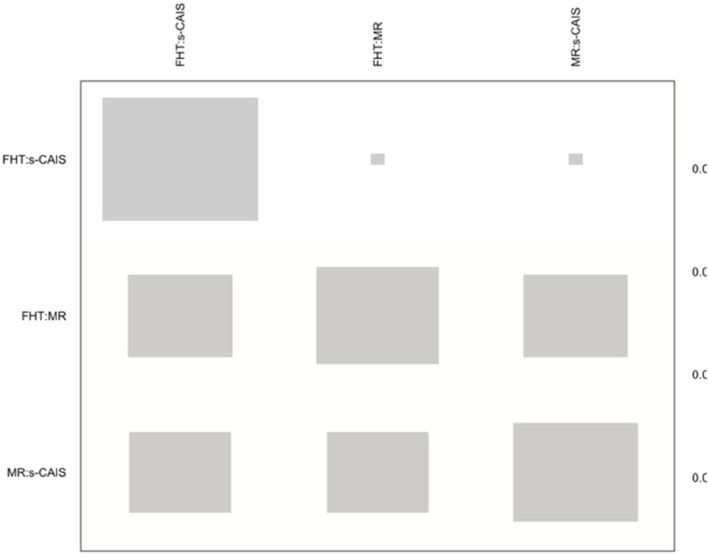
Net heat plot at the coronal entry-point. Gray boxes signify the importance of the direct estimate of one treatment comparison in the column to the network estimation of another treatment comparison in the row. Larger boxes indicate more important comparisons

NMA showed heterogeneity (within designs) by a Q test = 71.0 (*p*-value < 0.001), however inconsistency (between designs) was not detected by a Q test = 3.14 (*p*-value = 0.076). The net heat plot is a matrix visualization proposed by Krahn et al. (2013) [21] that highlights the contribution of the direct estimates to the network and detect possible sources of inconsistency. The size of the gray squares indicates the contribution of the direct estimates (shown in the column) to the network estimates (shown in the row). The colors are associated with the change in inconsistency when detaching one comparison from the network. Blue colors indicate an increase of inconsistency and warm colors indicate a decrease. The absence of color indicates that neither treatment comparison could be considered a source of inconsistency to the NMA (Fig. 4).

#### Apical deviation

**Fig. 5:**
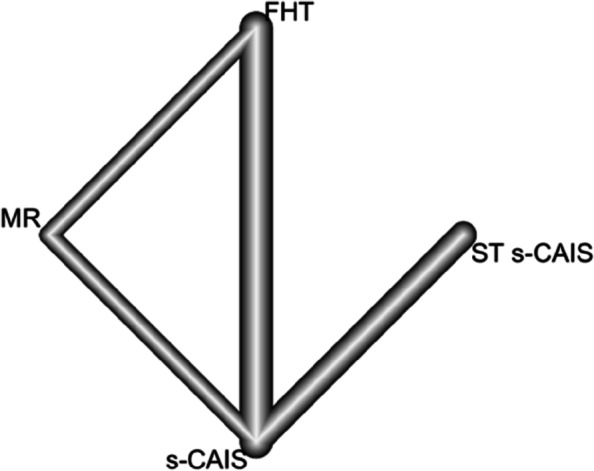
NETWORK plot of orthodontic mini-implant placement techniques at the apical end-point. Seven pairs of comparisons between four orthodontic mini-implant techniques and four direct comparisons included

Seven RETs (seven pairs of comparisons) were included in a frequentist NMA examining four orthodontic mini-implant placement techniques (s-CAIS, ST s-CAIS, MR and FHT) to analyze the accuracy of the orthodontic mini-implants placement techniques at the apical end-point. The data were combined with a random effects model. The nodes represent treatments, and the lines connecting the nodes are the four direct comparisons included in the NMA (Fig. 5).

**Fig. 6:**
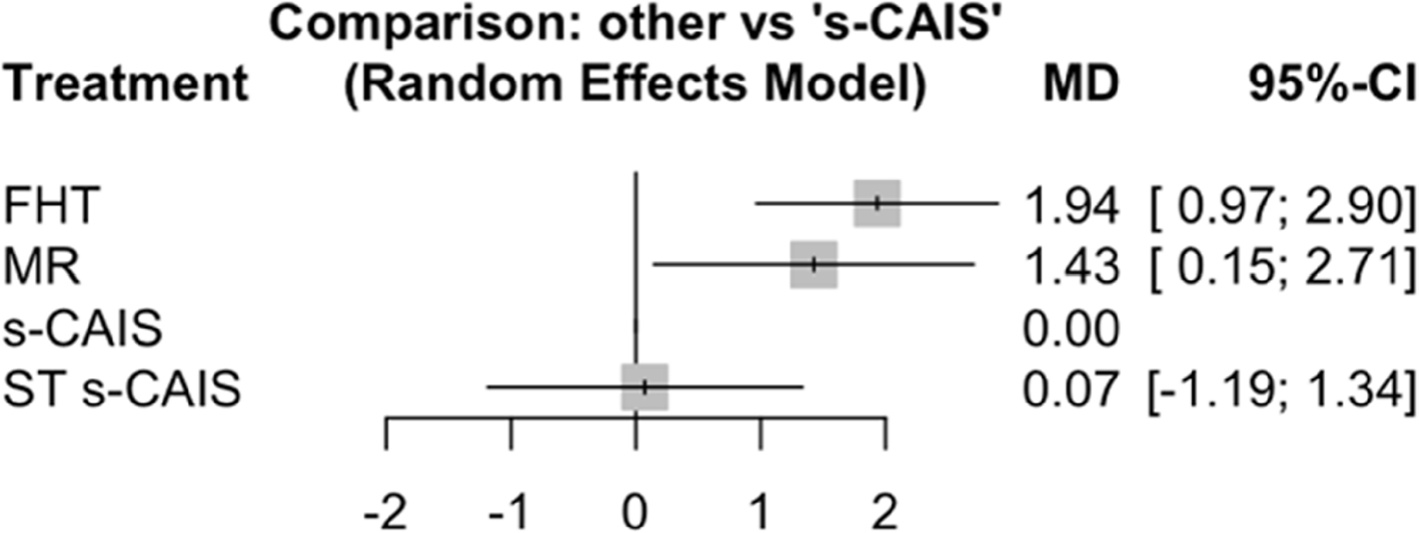
Forest plot of the mean differences (MD) between MR, s-CAIS and ST s-CAIS orthodontic mini-implants placement techniques respect the conventional free-hand technique at apical end-point

Mean differences between MR, s-CAIS and ST s-CAIS orthodontic mini-implants placement techniques and conventional free-hand technique were assessed at apical end-point. Taking as reference technique s-CAIS, the highest mean difference apical deviation was FHT (*p*-value < 0.001), followed by MR (*p*-value = 0.029) and ST s-CAIS (*p*-value = 0.908) (Fig. 6).

**Fig. 7:**
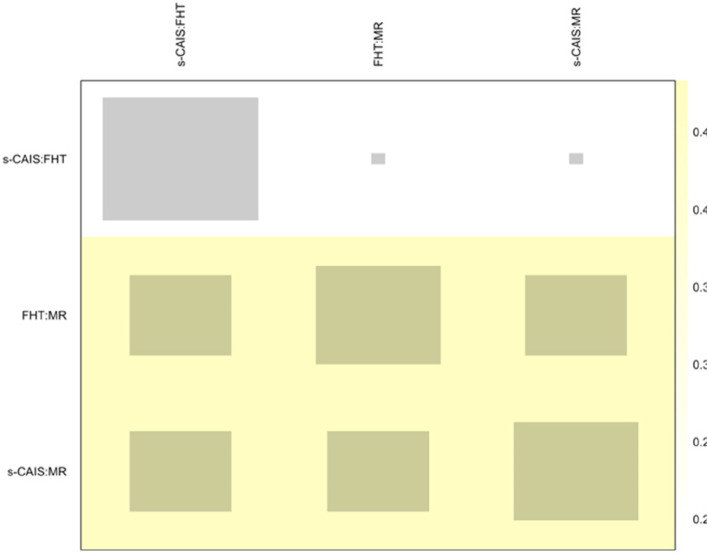
Net heat plot at the apical end-point

NMA showed heterogeneity (within designs) by a Q test = 90.2 (*p*-value < 0.001), however inconsistency (between designs) was not detected by a Q test = 0.22 (*p*-value = 0.641). The net heat plot detected slight changes (yellow colour) in consistency (not significant) when detaching any comparison from the network (Fig. 7).

#### Angular deviation

**Fig. 8:**
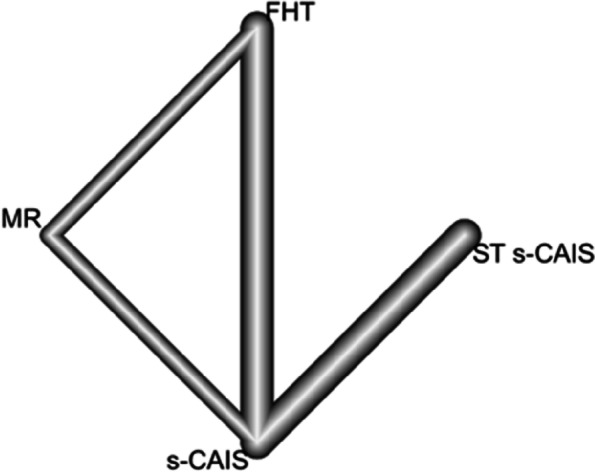
NETWORK plot of orthodontic mini-implant placement techniques at the angular deviation. Eight pairs of comparisons between four orthodontic mini-implant techniques and four direct comparisons included

Eight RETs (eight pairs of comparisons) were included in a frequentist NMA examining four orthodontic mini-implant placement techniques (s-CAIS, ST s-CAIS, MR and FHT) to analyze the accuracy of the orthodontic mini-implants placement techniques at the angular deviation. The data were combined with a random effects model. The nodes represent treatments, and the lines connecting the nodes are the four direct comparisons included in the NMA (Fig. 8).

**Fig. 9:**
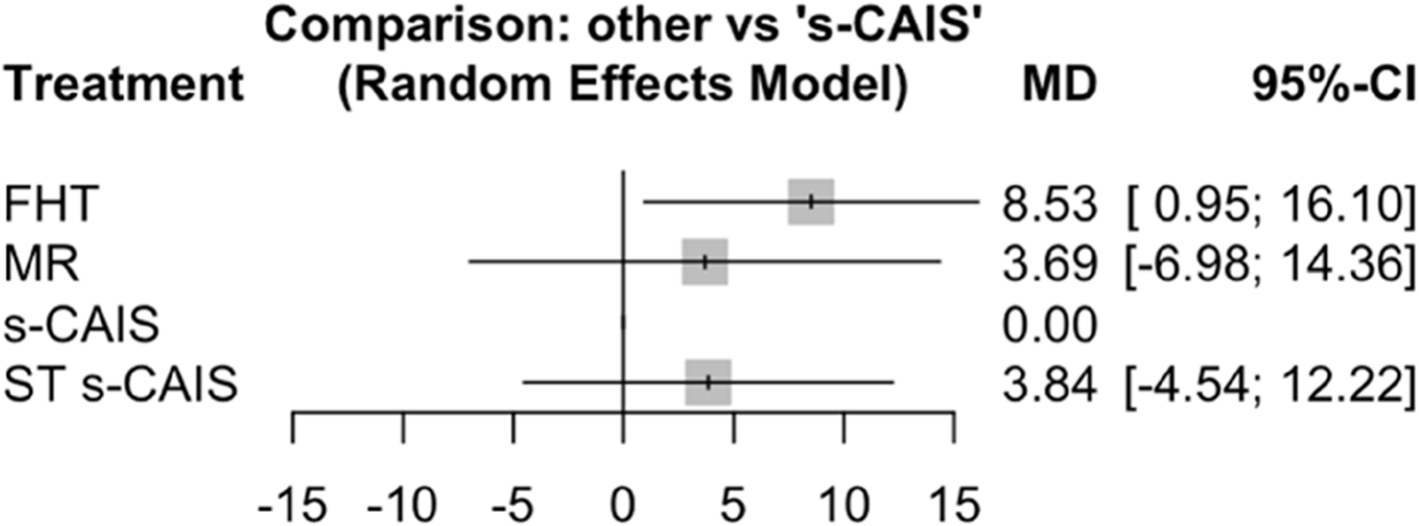
Forest plot of the mean differences (MD) between MR, s-CAIS and ST s-CAIS orthodontic mini-implants placement techniques respect the conventional free-hand technique at angular deviation

Mean differences between MR, s-CAIS and ST s-CAIS orthodontic mini-implants placement techniques and conventional free-hand technique were assessed at angular deviation. Taking as reference technique s-CAIS, the highest mean difference angular deviation was FHT (*p*-value = 0.027), followed by ST s-CAIS (*p*-value = 0.369) and MR (*p*-value = 0.498) (Fig. 9).

**Fig. 10:**
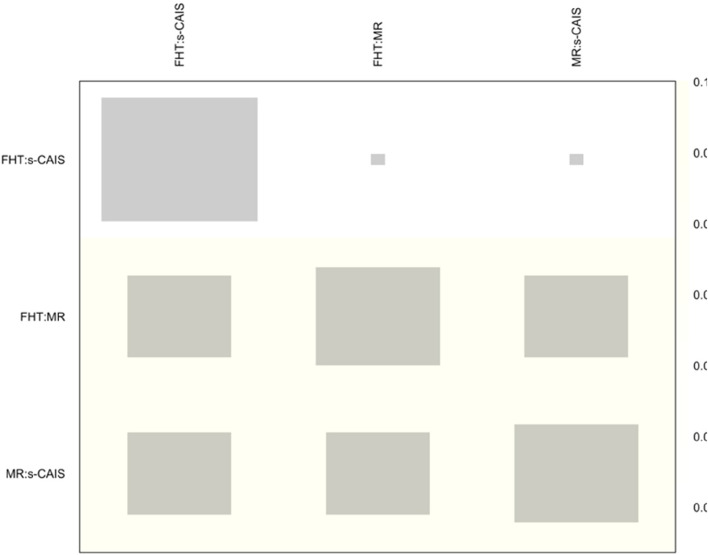
Net heat plot at the angular deviation

Heterogeneity (Q test = 321.7; *p* < 0.001) and inconsistency (Q test = 58,8; *p* < 0.001) were found in the NMA at the angular deviation. However, the net heat plot (Fig. 10) detected very slight changes in inconsistency when detaching any comparison from the network.

#### Ranking of the accuracy of the orthodontic mini-implant techniques

**Fig. 11:**
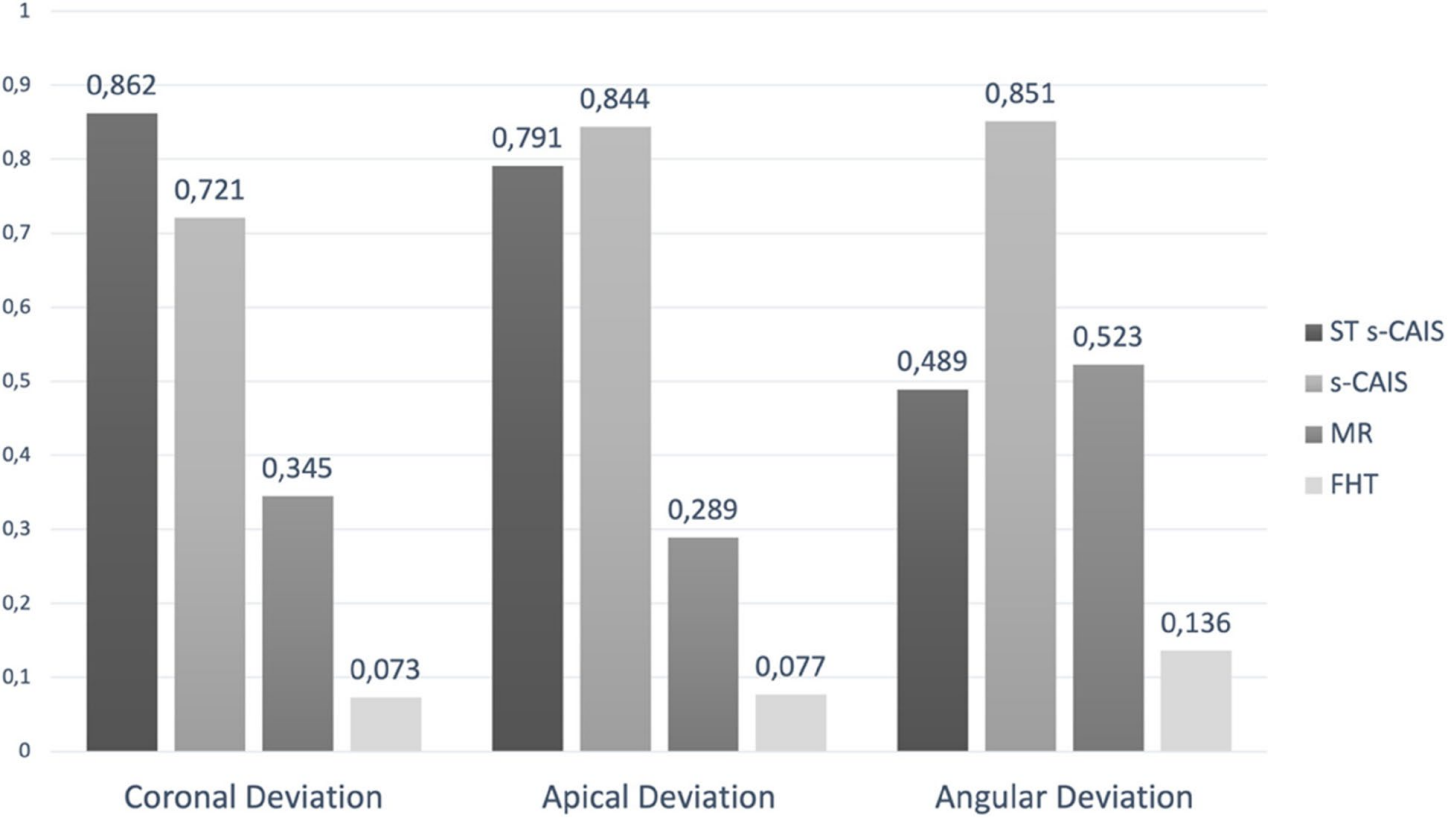
Ranking of the orthodontic mini-implant placement techniques and P-score

The ranking of the accuracy of the orthodontic mini-implant placement techniques was performed according to the P-score, which measures the degree of certainty and indicates whether one orthodontic mini-implant placement technique is superior to the others. The P-score is measured on a scale of 0 to 1. The higher P-score indicates desirable treatment compared to the other (Fig. 11). At the coronal deviation ST s-CAIS presented the highest P-score (0.862), followed by s-CAIS (0.721). At the apical deviation, s-CAIS presented the highest P-score (0.844), followed by ST s-CAIS (0.791). Finally, at the angular deviation s-CAIS presented the highest P-score (0.851), followed by MR (0.523) and ST-CAIS (0.489).

## Discussion

The objective of this systematic review and NMA was to analyze the accuracy of dental miniscrew placement when using surgical guides created with computer-aided design and computer-aided manufacturing techniques for the orthodontic mini-implants placed in the inter-radicular space.

Dynamic navigation (DN) has been used in several medical specialties among which are ophthalmology, otolaryngology, neurosurgery, or orthognathic surgery. Moreover, computer-aided navigation technique has already used in dental surgery to reduce deviations from the preoperative planned implant position. This technique has allowed to reduce clinical complications and making more favorable and predictable system [22]. However, Tao et al. showed statistically significant higher deviations (*p* < 0.001) of the dynamic navigation technique comparing to robotic systems [23]. Jorba- García et al. compared the dental implant placement accuracy between the computer-aided dynamic navigation and freehand techniques and reported that the dynamic navigation technique showed more accuracy at the global apex deviation (1.33 mm vs 2.26 mm) and angular deviation (1.6° vs 9.7°) [24]. In addition, Mediavilla Guzman et al. compared the accuracy of dental implant placement between computer-aided static implant surgery and computer-aided static implant surgery; however, no statistically significant differences were reported at the global apex deviation (1.20 mm vs 1.18 mm) and global coronal deviation (0.78 mm vs 0,85 mm) [25]. Additionally, Pellegrino et al. did not reveal statistically significant differences (*p* < 0.01) between the accuracy of dental implant placement between totally edentulous and partially edentulous patients using computer-aided static implant surgery [26]. Wei et al. also compared the accuracy of the dental implant placement using computer-aided dynamic navigation technique, showing less deviation angle in the maxilla (1.6° ± 1.27°) than mandible phantom (1.81° ± 1.28°) (*p* = 0.02); especially with the robotic system (0.87° ± 0.44° [27]. In addition, some studies have applied the computer-aided navigation technology to the location of root canals systems, to reduce the access and improve the prognosis for endodontically treated tooth [28, 29]. Moreover, Buchgreitz et al. highlighted the accuracy of endodontic access cavities and root canal system location in teeth with pulp canal obliteration using a guide rail based on CBCT scan and optical surface scan [30]. Likewise, Zehnder et al. found few deviations between planned and prepared endodontic access cavities and root canal system location (0.17 vs 0.47 mm) [31]. In conclusion, these findings have allow to improve the success rate and prognosis of root canal treatment.

The use of orthodontic mini-implants has been widely used to improve the anchorage in orthodontics; moreover, they have been also used for the treatment of extremely complex cases which used to be treated more invasively with surgery or extractions in the past [1]. In addition, orthodontic mini-implants have been also placed in the anterior palate for orthodontic anchorage. Iodice et al. reported that the use of surgical templates facilitates temporary anchorage devices insertion, allowing less-experienced clinicians to use palatal implants [32] .These results were aligned with those obtained by Migliorati et al. who highlighted the efficiency of the digital workflow [33]. In addition, Pozzan et al. reported statistically significant differences between the deviations of the digital placement technique and the deviations of the free-hand technique (*p* < 0.001) [34]. The results of the present NMA showed that the success of orthodontic mini-implant placement can be improved by computer-aided static navigation techniques. Specifically, the ST s-CAIS and s-CAIS showed less deviation values at the coronal entry-point deviation than the MR study group. These findings are aligned with those obtained by Qiu et al. that reported less coronal entry-point deviations using s-CAIS (0.15 ± 0.09 mm) than the free-hand conventional technique (0.48 ± 0.46 mm) [14]; Suzuki et al. also reported less coronal entry-point deviations using computer-aided static navigation techniques (1.0 ± 0.4 mm) comparing to the free-hand conventional technique [13]. Moreover, Riad Deglow et al. revealed less deviation values at the coronal entry-point attributed to the s-CAIS (1.06 ± 0.59 mm) and MR techniques (1.74 ± 0.52 mm) than the free-hand conventional technique (2.2 ± 2.0 mm) [19].

Furthermore, the results of the present NMA evidenced that the s-CAIS showed more accurate results at the apical end-point followed by the ST s-CAIS and MR techniques. In addition, Qiu et al. reported also less deviation values using computer-aided static navigation techniques (0.28 ± 0.23 mm) than the free-hand conventional technique (0.81 ± 0.61 mm) [14]; as well as the findings reported by Bae et al. (0.73 mm) [12]. In addition, the accuracy of the orthodontic mini-implant placement using muco-supported and dental-supported surgical templates have been also analysed. Kniha et al. reported higher deviation values at the apical end-point using the ST s-CAIS (1.91 ± 0.79 mm) respect the dental supported s-CAIS (1.77 ± 0.85 mm) [18].

Finally, the results of the present NMA evidenced that the s-CAIS showed the highest angular deviations followed by the MR and ST s-CAIS techniques. Additionally, Riad Deglow et al. analysed the accuracy of orthodontic mini-implant placement techniques using MR, showing more accurate results (5.55° ± 2.46°) than the free-hand conventional technique (7.58° ± 3.6°) [19]. However, Yu et al. did not reported statistically significant deviations between the planned and performed orthodontic mini-implants at angular level using s-CAIS (1.01° ± 7.25°) [15]. Moreover, Möhlhenrich et al. reported higher angular deviations between ST s-CAIS (6.46° ± 5.5°) and s-CAIS (3.67° ± 2.25°) [16].

Briefly, we can appreciate that the s-CAIS or ST s-CAIS orthodontic mini-implant placement technique improve the accuracy of the orthodontic mini-implant insertion, compared to the MR technique or the free-hand conventional technique. Riad Deglow et al. reported that the accuracy rate associate to the MR placement technique is sensitive to the operator's experience [19]. This is the main limitation of MR technique compared with s-CAIS techniques. Additionally, Bae et al. selected two operators with different levels of experience to analyse the sensitivity of the technique to operator's experience using FHT and s-CAIS to insert orthodontic mini-implants and reported that the differences between operators were higher in the FHT placement technique than in the s-CAIS placement technique [12]. Thus, s-CAIS placement technique should be recommended to operators without experience; however, it is mandatory to develop further studies to analyse the accuracy of s-CAIS and MR placement techniques.

Furthermore, root contact and the invasion of the maxillary sinus are considered the more common intraoperative complications during the orthodontic mini-implants placement; specially between the first and second upper premolar teeth [5]. Moreover, Motoyoshi et al. showed that the higher the diameter and length of the orthodontic mini-implant, the higher root damage risk [35]. However, root contact use to be superficial and the defects do not usually require treatment since they usually repair spontaneously after removing the orthodontic mini-implant [36].

These computer-aided innovative techniques are developed to minimize clinical complications. Kuroda et al. suggested that proximity to the root processes was the most relevant risk factor in the stability of orthodontic mini-implants [6]. Moreover, Qiu et al. reported ten injuries of the root processes using the FHT orthodontic mini-implant placement technique and no root damage using the s-CAIS orthodontic mini-implant placement technique [15]. However, Bae et al. showed 30% of root damage using the FHT orthodontic mini-implant placement technique and 16% s-CAIS orthodontic mini-implant placement technique [12]. These results suggest that the use of surgical templates can considerably reduce the risk of root perforation. In contrast, Kim et al. concluded that root proximity alone could not be considered a risk factor for mini-implant failure [37].

The use of surgical templates based on CBCT scans and mixed reality devices have been shown to successfully improve the accuracy of orthodontic mini-implants placement and reduce the risk of root damage. However, these orthodontic mini-implants placement techniques are not free of risks related to the thickness of the layers, or the size of the voxels, since a reduced voxel size improves the quality of the image but produces a greater amount of radiation [38]. In addition, the CBCT scan can be affected by the Hounsfield units, used to quantify the density or brightness value of the image. Pauwels et al. stated that CBCT manufacturers and software providers use Hounsfield values that are not always mutually valid [39]. Finally, the operator's experience and technique of the X-ray operator, as well as the position of the patient, uncontrolled mandibular movements or the presence of metallic objects that cause artifacts [40].

A limitation of this systematic review and network meta-analysis is the possibility that not all articles related to the selection criteria were identified, although the risk was decreased because three databases were searched. In addition, most of the studies showed a high methodological quality, according to the CRIS scale. Therefore, further, better designed clinical studies with higher quality are necessary.

## Conclusions

Within the limitations of this study, it was found that the image-guided-based orthodontic mini-implants placement techniques showed more accuracy than the free-hand conventional placement technique for the orthodontic mini-implants placed in the inter-radicular space; specially the computer-aided static navigation techniques.

## Data Availability

The datasets used and/or analyzed during the current study available from the corresponding author on reasonable request.

## References

[1] Kanomi R (1997). Mini-implant for orthodontic anchorage. J Clin Orthod.

[2] Reynders R, Ronchi L, Bipat S (2009). Mini-implants in orthodontics: a systematic review of the literature. Am J Orthod Dentofacial Orthop.

[3] Becker K, Pliska A, Busch C, Wilmes B, Wolf M, Drescher D (2018). Efficacy of orthodontic mini implants for en masse retraction in the maxilla: a systematic review and meta-analysis. Int J Implant Dent.

[4] Wilmes B, Olthoff G, Drescher D (2009). Comparison of skeletal and conventional anchorage methods in conjunction with pre-operative decompensation of a skeletal class III malocclusion. J Orofac Orthop.

[5] Suzuki EY, Buranastidporn B (2005). An adjustable surgical guide for miniscrew placement. J Clin Orthod.

[6] Kuroda S, Yamada K, Deguchi T, Hashimoto T, Kyung HM, Takano-Yamamoto T (2007). Root proximity is a major factor for screw failure in orthodontic anchorage. Am J Orthod Dentofacial Orthop.

[7] Wong JC, Palomo JM, Landers MA, Figueroa A, Hans MG (2008). Image quality produced by different cone-beam computed tomography settings. Am J Orthod Dentofacial Orthop.

[8] Banzi R, Moja L, Liberati A, Gensini GF, Gusinu R, Conti AA (2009). Measuring the impact of evidence: the Cochrane systematic review of organised stroke care. Intern Emerg Med.

[9] Krithikadatta J, Gopikrishna V, Datta M (2014). CRIS Guidelines (Checklist for Reporting In-vitro Studies): A concept note on the need for standardized guidelines for improving quality and transparency in reporting in-vitro studies in experimental dental research. J Conserv Dent.

[10] Freeman SC, Fisher D, White IR, Auperin A, Carpenter JR (2019). Identifying inconsistency in network meta-analysis: Is the net heat plot a reliable method?. Stat Med.

[11] Rücker G, Schwarzer G (2015). Ranking treatments in frequentist network meta-analysis works without resampling methods. BMC Med Res Methodol.

[12] Bae MJ, Kim JY, Park JT, Cha JY, Kim HJ, Yu HS, Hwang CJ (2013). Accuracy of miniscrew surgical guides assessed from cone-beam computed tomography and digital models. Am J Orthod Dentofacial Orthop.

[13] Suzuki EY, Suzuki B (2008). Accuracy of miniscrew implant placement with a 3-dimensional surgical guide. J Oral Maxillofac Surg.

[14] Qiu L, Haruyama N, Suzuki S, Yamada D, Obayashi N, Kurabayashi T, Moriyama K (2012). Accuracy of orthodontic miniscrew implantation guided by stereolithographic surgical stent based on cone-beam CT-derived 3D images. Angle Orthod.

[15] Yu JJ, Kim GT, Choi YS, Hwang EH, Paek J, Kim SH, Huang JC (2012). Accuracy of a cone beam computed tomography-guided surgical stent for orthodontic mini-implant placement. Angle Orthod.

[16] Möhlhenrich SC, Brandt M, Kniha K, Bock A, Prescher A, Hölzle F, Modabber A, Danesh G (2020). Suitability of virtual plaster models superimposed with the lateral cephalogram for guided paramedian orthodontic mini-implant placement with regard to the bone support. J Orofac Orthop.

[17] Möhlhenrich SC, Brandt M, Kniha K, Prescher A, Hölzle F, Modabber A, Wolf M, Peters F (2019). Accuracy of orthodontic mini-implants placed at the anterior palate by tooth-borne or gingiva-borne guide support: a cadaveric study. Clin Oral Investig.

[18] Kniha K, Brandt M, Bock A, Modabber A, Prescher A, Hölzle F, Danesh G, Möhlhenrich SC (2021). Accuracy of fully guided orthodontic mini-implant placement evaluated by cone-beam computed tomography: a study involving human cadaver heads. Clin Oral Investig.

[19] Riad Deglow E, Toledano Gil S, Zubizarreta-Macho Á, Bufalá Pérez M, Rodríguez Torres P, Tzironi G, Albaladejo Martínez A, López Román A, Hernández MS (2021). Influence of the Computer-Aided Static Navigation Technique and Mixed Reality Technology on the Accuracy of the Orthodontic Micro-Screws Placement. An In Vitro Study. J Pers Med.

[20] Lim JE, Lim WH, Chun YS (2008). Quantitative evaluation of cortical bone thickness and root proximity at maxillary interradicular sites for orthodontic mini-implant placement. Clin Anat.

[21] Krahn U, Binder H, König J (2013). A graphical tool for locating inconsistency in network meta-analyses. BMC Med Res Methodol.

[22] Mena-Álvarez J, Rico-Romano C, Lobo-Galindo AB (2017). Endodontic treatment of dens evaginatus by performing a splint guided access cavity. J Esthet Restor Dent.

[23] Tao B, Feng Y, Fan X, Zhuang M, Chen X, Wang F, Wu Y (2022). Accuracy of dental implant surgery using dynamic navigation and robotic systems: An in vitro study. J Dent.

[24] Jorba-García A, Figueiredo R, González-Barnadas A, Camps-Font O, Valmaseda-Castellón E (2019). Accuracy and the role of experience in dynamic computer guided dental implant surgery: An in-vitro study. Med Oral Patol Oral Cir Bucal.

[25] Mediavilla Guzmán A, Riad Deglow E, Zubizarreta-Macho Á, Agustín-Panadero R, Hernández MS (2019). Accuracy of Computer-Aided Dynamic Navigation Compared to Computer-Aided Static Navigation for Dental Implant Placement: An In Vitro Study. J Clin Med.

[26] Pellegrino G, Ferri A, Del Fabbro M, Prati C, Gandolfi MG, Marchetti C (2021). Dynamic Navigation in Implant Dentistry: A Systematic Review and Meta-analysis. Int J Oral Maxillofac Implants.

[27] Wei SM, Zhu Y, Wei JX, Zhang CN, Shi JY, Lai HC (2021). Accuracy of dynamic navigation in implant surgery: A systematic review and meta-analysis. Clin Oral Implants Res.

[28] Plotino G, Grande NM, Isufi A (2017). Fracture Strength of Endodontically Treated Teeth with Different Access Cavity Designs. J Endod.

[29] Ikram OH, Patel S, Sauro S (2009). Micro-computed tomography of tooth tissue volume changes following endodontic procedures and post space preparation. Int Endod J.

[30] Buchgreitz J, Buchgreitz M, Mortensen D, Bjørndal L (2016). Guided access cavity preparation using cone-beam computed tomography and optical surface scans - an ex vivo study. Int Endod J.

[31] Zehnder MS, Connert T, Weiger R, Krastl G, Kühl S (2016). Guided endodontics: accuracy of a novel method for guided access cavity preparation and root canal location. Int Endod J.

[32] Iodice G, Nanda R, Drago S, Repetto L, Tonoli G, Silvestrini-Biavati A, Migliorati M (2022). Accuracy of direct insertion of TADs in the anterior palate with respect to a 3D-assisted digital insertion virtual planning. Orthod Craniofac Res.

[33] Migliorati M, Drago S, Pozzan L, Contardo L (2022). Does the planned miniscrew position reflect the achieved one? A clinical study on the reliability of guided miniscrew insertion using lateral cephalogram and maxillary stereolithography file for planning. Am J Orthod Dentofacial Orthop.

[34] Pozzan L, Migliorati M, Dinelli L, Riatti R, Torelli L, Di Lenarda R, Contardo L (2022). Accuracy of the digital workflow for guided insertion of orthodontic palatal TADs: a step-by-step 3D analysis. Prog Orthod.

[35] Motoyoshi M, Matsuoka M, Shimizu N (2007). Application of orthodontic mini-implants in adolescents. Int J Oral Maxillofac Surg.

[36] Asscherickx K, Vannet BV, Wehrbein H, Sabzevar MM (2005). Root repair after injury from mini-screw. Clin Oral Implants Res.

[37] Kim SH, Kang SM, Choi YS, Kook YA, Chung KR, Huang JC (2010). Cone-beam computed tomography evaluation of mini-implants after placement: Is root proximity a major risk factor for failure?. Am J Orthod Dentofacial Orthop.

[38] Jacobs R, Quirynen M (2014). Dental cone beam computed tomography: justification for use in planning oral implant placement. Periodontol 2000.

[39] Pauwels R, Jacobs R, Bosmans H, Pittayapat P, Kosalagood P, Silkosessak O, Panmekiate S (2014). Automated implant segmentation in cone-beam CT using edge detection and particle counting. Int J Comput Assist Radiol Surg.

[40] Bornstein MM, Horner K, Jacobs R (2017). Use of cone beam computed tomography in implant dentistry: current concepts, indications and limitations for clinical practice and research. Periodontol 2000.

